# The mental health of asylum seekers in Australia and the role of psychiatrists

**DOI:** 10.1192/bji.2018.11

**Published:** 2018-08

**Authors:** Derrick Silove, Sarah Mares

**Affiliations:** 1 Scientia Professor, School of Psychiatry, University of New South Wales, Sydney, Australia; 2 Conjoint Senior Lecturer, School of Psychiatry, University of New South Wales, Sydney, Australia. Email s.mares@unsw.edu.au; 3 PhD candidate, Flinders University, Adelaide, Australia

**Keywords:** Ethics, human rights, immigration detention, mental health, asylum seekers and refugees, psychiatry

## Abstract

There are more displaced people around the world than ever before, and over half are children. Australia and other wealthy nations have implemented increasingly harsh policies, justified as ‘humane deterrence’, and aimed at preventing asylum seekers (persons without preestablished resettlement visas) from entering their borders and gaining protection. Australian psychiatrists and other health professionals have documented the impact of these harsh policies since their inception. Their experience in identifying and challenging the effects of these policies on the mental health of asylum seekers may prove instructive to others facing similar issues. In outlining the Australian experience, we draw selectively on personal experience, research, witness account issues, reports by human rights organisations, clinical observations and commentaries. Australia’s harsh response to asylum seekers, including indefinite mandatory detention and denial of permanent protection for those found to be refugees, starkly demonstrates the ineluctable intersection of mental health, human rights, ethics and social policy, a complexity that the profession is uniquely positioned to understand and hence reflect back to government and the wider society.

The Office of the United Nations High Commissioner for Refugees estimates the global number of refugees to stand at an unprecedented 65.3 million, with 50% being children (UNHCR, 2017). Most receive sanctuary in neighbouring countries, a small percentage reaching North America, Europe and Australasia. Despite this, Australia and other wealthy nations have implemented increasingly harsh policies of so-called ‘humane deterrence’, aimed at preventing asylum seekers (persons without preestablished resettlement visas) from entering their borders.

The experiences of Australian psychiatrists and allied health professionals in confronting the mental health effects of these policies on asylum seekers may prove instructive to colleagues in other countries facing similar issues. In outlining the Australian experience, we draw selectively on personal experience, research, witness accounts, reports by human rights organisations, clinical observations and commentaries.

## Australia's history of migration

Australia was invaded and colonised by the British around 1788 and is now a diverse multicultural nation. Apart from general immigrants, Australia ranks high in per capita intake of refugees accepted for resettlement overseas, that is, the off-shore program (RCA, 2015). Until the 1970s, this conformed to the White Australia policy, when a political transformation led to the acceptance of substantial numbers of South-east Asian refugees who were provided with unrestricted rights to education, work, income support, healthcare and citizenship. The enlightened nature of this resettlement policy is likely to have contributed to the remarkably sound mental health outcomes recorded for the Vietnamese two decades later (Steel *et al*, 2002).

## Policies of deterrence

The late 1980s was a critical turning point politically as increasing numbers of asylum seekers arrived by boat on Australia's northern coast. In 1992 the government ushered in a two-tier policy: Australia accepts between 12 000 and 20 000 ‘off-shore’ refugees screened overseas and supported in resettlement. In parallel, an increasingly harsh policy of deterrence applies in relation to asylum seekers, particularly those arriving by boat. Although policy has varied, there has been increasing reliance on mandatory indefinite detention applied to particular categories of asylum seekers. Many adults and children have been held for long periods in remote, prison-like detention centres and, more recently, on pacific island nations north of Australia. Other asylum seekers reside temporarily in the community under restrictive conditions, with limited rights to work, healthcare, education and family reunion. The details and harshness of these policies have fluctuated in step with prevailing political and public opinion.

Since 2013, a policy has been in place mandating the transfer of all sea arrivals to the island of Nauru (one of the smallest, least populous and under-resourced nations in Oceania) and Manus Island in Papua New Guinea. Many detainees have been held for over 4 years despite incidents of violence, abuse and self-harm, including violent deaths of detainees. The policy has remained steadfast, the prohibition against any detainee ever settling in Australia remaining in place, despite over 90% of detainees being identified as legitimate refugees after rigorous screening. A turning point came with the judicial ruling in Papua New Guinea that the Manus Island detention centre was illegal, leading to a hasty closure of the facility. This action provoked a humanitarian crisis in late 2017, when 600 inmates refused to leave, preferring to live without water, food and services than to be resettled in the general island community where, after prior incidents of violence directed at them, they feared for their safety. Nevertheless, the closure has generated some impetus to arrange resettlement of detainees from Nauru in other countries.

## Roles of psychiatrists and allied health professionals

For 25 years, Australia psychiatrists and allied health colleagues have played important roles in responding to the treatment of asylum seekers.

### Identifying the risks and awareness raising

From the outset, reports and commentaries by psychiatrists and other health professionals drew attention to the potential re-traumatising effects of detention and other restrictive policies on a population exposed to prior persecution and mass violence (Silove *et al*, 1993), Insider testimony, including from a detained doctor, provided support for these assertions. Since that time, psychiatrists have remained prominent in raising concerns and providing expert testimony about the mental health effect of the detention policy in the media, with the issue drawing national and worldwide attention through editorials in major international journals (e.g. Silove *et al*, 2001).

### Documentation and research

There are significant challenges in undertaking research in this field, including gaining informed consent and other ethical constraints, access to asylum seekers, representativeness of samples and transcultural and language issues in assessment and measurement.

Despite this, research was initiated among adult asylum seekers soon after restrictive policies were implemented. The findings paint a consistent picture of markedly elevated rates of mental distress (including symptoms of post-traumatic stress, depression and anxiety) among asylum seekers compared with compatriot refugees with permanent residency status. In addition, the dual experience of detention and release on temporary protection visas was found to be associated with persisting traumatic stress symptoms and functional impairment (Steel *et al*, 2006).

Despite formidable obstacles in access, initial observations of children in remote Australian detention centres (Mares *et al*, 2002) and mixed method studies (Mares & Jureidini, 2004; Steel *et al*, 2004) converged to reveal extraordinarily high rates of a wide range of psychiatric disorders in children and their parents (Mares, 2016). This accruing body of evidence, buttressed by data collected under the authorities' own auspices (Young & Gordon, 2016), has played a discernible role in changing government responses over time. From a position of denial of the mental health harm being done and/or dismissing or denigrating the ‘messengers’, the tendency now is to tacitly accept and justify the duress caused in terms of protecting borders and humane deterrence; that is, the saving of lives following drownings of asylum seekers at sea. The evidence accrued in Australia is summarised in Table 1.

**Table 1:** Asylum policy and mental health; principles derived from accrued evidence

1.	Successful adaptation and resettlement of refugees is supported by post arrival access to education, health and language services and pathways to citizenship.
2.	Refugee mental health is undermined by post-migration stressors, in particular prolonged immigration detention and temporary protection.
3.	Mental health is significantly worsened in asylum seekers who experience prolonged detention compared with those never detained.
4.	Detained children are exposed to multiple and cumulative risks with substantial negative effects on health, development and family functioning.
5.	Rates of mental illness in detained adults and children resemble clinical populations and morbidity increases with length of time detained.

### Expert assessments

Evidence provided by psychiatrists, other health professionals and lawyers has proven pivotal in a series of inquiries into the effect of asylum policies by human rights groups, including the Australian Human Rights Commission and the United Nations Rapporteur on Human Rights (HREOC, 2004; AHRC, 2014). Mental health professionals and lawyers developed comprehensive protocols and training materials to assist the comprehensive assessment of refugee claims to limit risk of distorted testimonies, which result in erroneous decision-making. Among the factors that require sensitive consideration are risk of cultural and linguistic misunderstandings, the effect of post-traumatic stress disorder and depressive symptoms on the capacity to provide a coherent narrative, the importance of not overlooking the effect of past head injury on cognition and memory, eliciting histories of politically motivated sexual abuse and recognising reticence arising from underlying fears of reprisal against the self and the family.

### Forging collaborations

Psychiatrists assumed leadership roles in forging collaborative networks within medical and allied professional groups. This strengthened the authority of these coalitions in attempts to influence policy. The solidarity achieved among diverse groups was unprecedented in Australia, particularly in the pursuit of a single but politicised health issue.

### Risks and costs

Colleagues have taken contrasting positions on the ethical challenges involved in this highly politicised work (Newman, 2016). Attempts to collaborate with government on asylum issues have largely failed. Senior psychiatrists who initially contributed to a detention health advisory committee ultimately determined that the risks of unintended collusion outweighed potential gains. Employees of private health providers in detention centres, including individual psychiatrists and other colleagues, continue to speak out against the compromised care and deleterious effect of conditions in detention, risking potential prosecution (Dudley, 2016). In response, the Royal Australian and New Zealand College of Psychiatrists recently updated its guidelines for psychiatrists working in Australian immigration detention centres.

Attempts have been made to undermine the veracity of research findings and public testimony of medical experts, including psychiatrists (see Maglen, 2007), and to discourage psychiatrists and allied professionals from pursuing research in this area by refusing access or making access difficult. Until recently, legislation made it an offence for a range of professionals to divulge any information based on their observations while working in detention centres (Dudley, 2016).

Individual psychiatrists have challenged the ethics of unconventional approaches to obtaining research data in this field, the two sides of the debate being aired in an issue of a bioethics journal devoted to the topic (Minas, 2004). Despite detractors, the urgency of undertaking research and the risk of silence on this topic was supported by both local and international colleagues (e.g. Kirmayer *et al*, 2004). A summary of the lessons learned is provided in Table 2.

**Table 2:** Lessons from the experiences of psychiatrists working with asylum seekers in Australia

1.	The health and mental health of people who seek asylum cannot be considered in isolation from broader social and political factors.
2.	Immigration detention illustrates the intersection of human rights and mental health, leading to an overlap in roles of clinician, researcher and advocate.
3.	It is almost impossible to undertake studies with detained populations in conventional ways. The results invariably will be contentious and politicised. Nonetheless, research into the effects of restrictive government policies should be supported, and pressure brought to bear to allow access to representative samples without risk to investigators or participants.
4.	Health professionals working within the Australian immigration detention system face major ethical challenges. It is a system that causes demonstrable harm and lacks independent oversight and transparency.
5.	Clinicians and researcher in this area require the ongoing support of colleagues and professional bodies.

## Conclusion

Psychiatrists and allied professionals have played a sustained role in garnering and publicising evidence of the mental health consequences of Australia's harsh immigration policies. The evidence is clear: restrictive policies, particularly prolonged immigration detention, are detrimental to the mental health of adult and child asylum seekers. It is of particular importance that psychiatrists have raised concerns and generated evidence soon after implementation of restrictive policies, undermining government claims of ignorance of the harm done by continuing these harsh policies over subsequent decades.

Over time, the effects of detention on children have proved most persuasive in swaying public opinion. Although few children remain in detention (some are held on Nauru), many thousands remain in a state of limbo in the community either on temporary visas or community variants of detention, and the restrictive policies applied to children seeking asylum remain.

Unsurprisingly, commitment to this area of public policy and human rights comes at a cost to those involved. The evidence has been variously challenged, denied, undermined, ignored or justified. Health professionals must grapple with the unresolvable dilemma of a commitment to assisting detained asylum seekers while simultaneously recognising the ethical and professional compromises inherent in working within a detention regime that lacks independent scrutiny or oversight and demonstrably creates the conditions that cause the very harms that mental health professionals aim to prevent and remediate.

Despite the challenges, we maintain that it is the core business of psychiatrists to document, research and bear witness to the consequences of social policies that undermine the mental health of vulnerable populations. The billions of dollars expended on Australia's detention regime would be better spent on resourcing effective preventative and therapeutic interventions for displaced and traumatised people. Australia's policy and practise of indefinite mandatory detention of asylum seekers starkly demonstrates the ineluctable intersection of mental health, human rights and ethics, and social policy, a complex maze that the profession is uniquely positioned to understand and hence reflect back to both governments and the wider society.
